# Predictive factors of radioiodine ablation success: results from a MEDIRAD prospective clinical study for thyroid cancer

**DOI:** 10.1530/ETJ-25-0097

**Published:** 2025-07-01

**Authors:** Jan Taprogge, Iain Murray, Hannah Sharman, Paul Gape, Francesca Leek, Carla Abreu, Lenka Vávrová, Kate Newbold, Kee H Wong, Markus Luster, Frederik A Verburg, Tino Schurrat, Lavinia Vija, Frédéric Courbon, Delphine Vallot, Manuel Bardiès, Sarah Schumann, Uta Eberlein, Michael Lassmann, Glenn Flux

**Affiliations:** ^1^ National Radiotherapy Trials Quality Assurance (RTTQA) Group, Joint Department of Physics, Royal Marsden NHSFT, Sutton, United Kingdom; ^2^ The Institute of Cancer Research, London, United Kingdom; ^3^ Joint Department of Physics, Royal Marsden NHSFT, Sutton, United Kingdom; ^4^ Thyroid Unit, Royal Marsden NHSFT, Sutton, United Kingdom; ^5^ Department of Nuclear Medicine, Philipps-University Marburg, Marburg, Germany; ^6^ Erasmus Medical Center, Department of Radiology and Nuclear Medicine, Rotterdam, Netherlands; ^7^ IUCT Oncopole, Toulouse, France; ^8^ Centre de Recherches en Cancérologie de Toulouse, UMR 1037, INSERM Université Paul Sabatier, Toulouse, France; ^9^ Institut de Recherches en Cancérologie de Montpellier, UMR 1194, INSERM Université de Montpellier, Montpellier, France; ^10^ Department of Nuclear Medicine, University Hospital Würzburg, Würzburg, Germany

**Keywords:** multi-centre trial, dosimetry, radioiodine, differentiated thyroid canc

## Abstract

**Objective:**

Serum thyroglobulin measurements are used in the long-term management of patients with differentiated thyroid cancer following thyroidectomy and radioiodine therapy. The use of predictive biomarkers, such as post-operative stimulated thyroglobulin levels and absorbed dose, may help to identify patients at risk of disease recurrence or an unsuccessful initial treatment.

**Methods:**

Differentiated thyroid cancer patients treated with 1.1 or 3.7 GBq of radioiodine using recombinant human thyrotropin stimulation or thyroid hormone withdrawal were recruited into observational clinical studies in France, Germany and the UK with aligned study endpoints (MEDIRAD). The maximum absorbed dose to the thyroid remnant was determined and compared to post-operative stimulated thyroglobulin with respect to its ability to predict ablation success. Radioiodine therapy success was defined as unstimulated or stimulated thyroglobulin level of <0.2 or <1.0 ng/mL 9–12 months post-radioiodine.

**Results:**

Ninety-four patients had follow-up data and negative antithyroglobulin antibody tests. Seventy-eight patients (83%) were deemed excellent biochemical responders. Post-operative thyroglobulin and maximum absorbed dose predicted ablation success with receiver operating characteristic area under the curves of 0.83 ± 0.05 (*P* < 0.001) and 0.64 ± 0.08 (*P* = 0.12). A dose–response relationship between maximum absorbed dose and ablation success was found for patients with a post-operative stimulated thyroglobulin of ≥1 ng/mL.

**Conclusions:**

Predictions of ablation success using post-operative stimulated thyroglobulin or the absorbed dose to the thyroid remnant could inform personalisation of management of differentiated thyroid cancer and identify patients where further treatments or more intensive follow-up are required. Patients with a post-operative stimulated Tg of <1 ng/mL likely do not benefit from radioiodine.

## Introduction

Differentiated thyroid carcinoma (DTC) has a good prognosis, with 5-year disease-specific survival rates of >90% for both papillary and follicular subtypes (1). Follow-up protocols in DTC focus on early detection of recurrence to guide and inform additional treatments and intensity of follow-up. Thyroglobulin (Tg) is an important biomarker for follow-up after thyroidectomy with or without radioiodine treatment (2, 3). Post-operative Tg levels, measured before radioiodine ablation, have also been shown to be predictive of clinical outcomes in DTC patients (4, 5, 6), but cut-off values vary between studies, and the presence of antithyroglobulin antibodies may limit the predictive accuracy (7).

A review of whether the success of ablation is dependent on the activity of radioiodine administered found that results were inconclusive (8). The absorbed dose delivered, defined as the amount of energy deposited per unit mass to thyroid remnants, has been hypothesised to be predictive of ablation success in single-centre studies, but further work in a multi-centre setting is required (9, 10, 11, 12). The absorbed dose can be calculated from a series of quantitative single-photon emission computed tomography (SPECT) scans to determine the total number of radioactive decays, the volume of the thyroid remnant and a conversion factor for mean absorbed dose per unit of activity. A potential drawback of thyroid remnant dosimetry is the limited accuracy with which the thyroid remnant volume can be assessed using SPECT, ultrasound, CT or MRI. Therefore, the absorbed dose delivered to the voxel with maximum uptake was calculated, as the volume is well defined, following methodology developed previously (9).

We report here results from a prospective multinational multi-centre clinical study, conducted as a work package within the Horizon 2020 MEDIRAD project (13), to investigate quantitative imaging and dosimetry in patients with DTC treated with radioiodine (see Supplementary Table 1 (see section on Supplementary materials given at the end of the article)). The main aim of this work package was to implement multi-centre quantitative imaging and dosimetry and to develop methodologies for future large-scale epidemiological studies (13). A secondary endpoint was to investigate potential biomarkers, including absorbed dose and post-operative Tg levels, as potential predictors of successful radioiodine ablation of DTC following thyroidectomy.

## Materials and methods

Three prospective observational studies were performed in four centres in three countries (University Hospital of Marburg (UMR) Germany, University Hospital Würzburg (UKW) Germany, Institut Universitaire du Cancer de Toulouse (IUCT-O) France and Royal Marsden Hospital NHS Foundation Trust (RMH) United Kingdom). Inclusion criteria and trial endpoints were aligned for each study. Ethical approval was obtained from the respective national and institutional review boards (see Supplementary Table 1). The study at RMH (ClinicalTrials.gov: NCT04391244) was approved by the East Midlands – Nottingham 1 Research Ethics Committee (20/EM/0022) and the institutional review board at the Royal Marsden Hospital. The common study protocol was presented to the local ethics committees at the Medical Faculty of the University of Würzburg (UKW) and Marburg (UKM) and approved (UKW: Az. 246/18, UKM: Az. 83/19), and was registered on EudraCT as 2019-002244-25. The study at IUCT-O was approved by the French national ethics committee (ID RCB: 2019-A01734-53). All patients provided written informed consent before registration.

Each centre was set up for standardised quantitative imaging. Details of standardised image acquisition and reconstruction protocols have been published previously (14).

### Patient inclusion criteria

Patient inclusion criteria were histologically proven DTC with near-total or staged thyroidectomy (hemithyroidectomy followed by completion thyroidectomy), aged 18 years or older. Patient exclusion criteria were a history of prior therapeutic radiation or recent radionuclide exposure, and medication or contrast agents known to interfere with iodine kinetics administered within the last 3 months, recent prior diagnostic radioiodine scan, external beam radiotherapy or systemic chemotherapy.

### I-131 administration and imaging schedule

Patients were administered either 1.1 or 3.7 GBq of radioiodine following thyroid-stimulating hormone (TSH) stimulation using either recombinant human thyrotropin (rhTSH) or thyroid hormone withdrawal, according to local protocols. Routine thyroid function tests, including post-operative stimulated Tg (hereafter referred to as Tg_stim,pre-I131_), TSH and antithyroglobulin antibody (Tg-Ab), were performed after TSH stimulation, before radioiodine.

One to six SPECT or SPECT/CT scans were performed for each patient between 6 and 168 h post-administration of radioiodine, based on local availability of resources and COVID restrictions. If only a single scan was acquired, this was performed between 24 and 96 h after administration. Details of the acquisition protocols used can be found in Supplementary Table 2.

### Dosimetry calculations

Thyroid remnant absorbed dose calculations were performed at the RMH using in-house developed dosimetry software. Analysis of thyroid remnant absorbed doses was performed for three participating clinical imaging centres for which thyroid remnant activity retention data were available: UMR, IUCT-O and RMH. Image data were reconstructed (see Supplementary Table 3 for details) and quantified using the system–volume calibration factors reported previously (14). Time-integrated activity was determined assuming a single exponential decay. For single time-point dosimetry, which only included centres performing rhTSH stimulation, an effective half-life of 68 h was used for the thyroid remnant, following the results of a previous study (15). Absorbed doses were calculated for the voxel with maximum time-integrated activity (D_max_ in Gy), following methodology previously presented (9), due to the uncertainty in the delineation of the thyroid remnant volume on the anatomical CT images. A matrix size of 128 × 128 was used for all SPECT scans, resulting in voxel sizes of (4.8)^3^ mm^3^ and (4.4)^3^ mm^3^ for Siemens and GE systems, respectively. A density of 1 g/cm^3^ was assumed, and absorbed dose calculations were performed assuming local energy deposition, taking into account the electron contribution to the absorbed dose only.

### Ablation outcome assessment

Routine follow-up Tg measurements (hereafter referred to as Tg_post-I131_), performed with a sensitive Tg assay (minimum detection limit: ≤0.2 ng/mL) or after TSH stimulation between 9 and 12 months following administration of radioiodine, were used to assess biochemical outcome using the cut-off values recommended in ATA guidelines (2), as shown in Table 1. Ablation failure was defined as (biochemical) incomplete or (biochemical) indeterminate response, while ablation success was defined as (biochemical) excellent response. No imaging data were collected as part of the follow-up in the present study, and response was, therefore, solely based on biochemical response.

**Table 1:** Biochemical outcome definitions using the cut-off values defined in the ATA guidelines (2).

Response	Routine follow-up Tg measurement results
Excellent response	Suppressed Tg < 0.2 ng/mL or
	TSH-stimulated Tg < 1 ng/mL
Indeterminate response	Suppressed Tg ≤ 0.2 to 1 ng/mL or
	TSH-stimulated Tg ≤ 1 to < 10 ng/mL
Incomplete response	Suppressed Tg ≥ 1 ng/mL or
	TSH-stimulated Tg ≥ 10 ng/mL

### Statistical analysis

Spearman’s rank correlation coefficient was calculated to assess correlations between the maximum absorbed dose to the thyroid remnant and post-operative Tg. Receiver operating characteristic (ROC) curve analysis was performed to assess the accuracy of using post-operative stimulated Tg levels (Tg_stim,pre-I131_) or maximum absorbed dose D_max_ as predictors of ablation success, defined here as excellent response. Patients were excluded from the analysis if either Tg_stim,pre-I131_ or D_max_ was missing, or if the Tg-Ab measurement was positive (centrally assessed by a consultant clinical oncologist based on the thyroid function test results, including Tg-Ab measurements and the normal ranges provided by each centre), and therefore indicating potential interference between Tg-Ab and Tg measurements. A sub-analysis was performed for patients with a Tg_stim,pre-I131_ above 1 ng/mL to exclude patients who had already reached the threshold for biochemical excellent response before radioiodine treatment.

Dose–response curves were fitted based on a two-parameter log-logistic model. For details, see Supplementary Table 4.

All statistical tests were exploratory, and testing was performed at the two-sided 5% significance level. Statistical analysis was performed using GraphPad Prism version 10.4.1 for Windows (GraphPad Software, USA).

## Results

### Patient characteristics

One hundred and six patients were recruited. Two patients (treated with 3.7 GBq) were lost to follow-up, and one patient withdrew from the trial; these were excluded from the analysis. Table 2 provides a summary of the characteristics of the included 103 patients before radioiodine therapy. A further nine patients were excluded from the predictive biomarker analysis due to positive Tg-Ab measurements, which could indicate potential interference between Tg-Ab and Tg. All patients were treated according to local standard of care, with three centres routinely administering 3.7 GBq, while at one centre patients received either 1.1 or 3.7 GBq. A single patient was treated with 2.5 GBq based on a local clinical decision.

**Table 2:** Patient characteristics of the study participants for whom follow-up data were available (*n* = 103) at four MEDIRAD WP3 centres using the 7th edition of the AJCC TNM staging. Data are presented as *n* (%), mean ± SD or as median (range).

Characteristics	Values
Age, years	47.6 ± 15.5
Female	77 (74.8)
Male	26 (25.2)
Histological subtype	
Papillary	85 (82.5)
Follicular	15 (14.6)
Mixed	3 (2.9)
Primary tumour and node stage	
T1Nx	17 (16.5)
T1N0	16 (15.5)
T1N1a	15 (14.6)
T1N1b	5 (4.9)
T2Nx	9 (8.7)
T2N0	13 (12.6)
T2N1a	8 (7.8)
T2N1b	1 (1.0)
T3Nx	5 (4.9)
T3N0	7 (6.8)
T3N1a	5 (4.9)
T3N1b	2 (1.9)
Prescribed RAI activity	
1,100 MBq	12 (11.7)
2,500 MBq	1 (0.9)
3,700 MBq	90 (87.4)
Stimulation protocol used	
THW*	20 (19.4)
rhTSH	83 (80.6)
Post-operative stimulated thyroid function tests	
TSH (mU/L)	109.7 ± 64.9
FT4 (pmol/L)	14.0 ± 8.2
FT3 (pmol/L)	5.5 ± 3.7
Tg (ng/mL)	1.9 (0.1–148.7)

* All THW patients were treated with 3.7 GBq.

### Patient outcomes

Of the 94 patients for whom follow-up data were available and who had negative Tg-Ab tests, five (5.3%) patients were classed as having incomplete response at 9–12 months after initial radioiodine treatment. Eleven (11.7%) and 78 (83.0%) of the patients showed indeterminate and excellent response, respectively. For the further analysis, the 16 patients with incomplete or indeterminate response and the 78 patients with excellent response were assigned to the ablation failure and ablation success groups, respectively.

### Maximum-voxel absorbed dose

**Figure 1:**
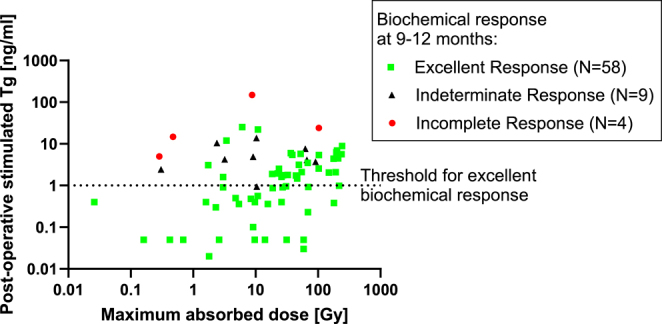
Post-operative stimulated Tg measurements of patients (*n* = 71) as a function of the maximum-voxel absorbed dose. Patients with incomplete, excellent and indeterminate response at 9–12 months post-radioiodine treatment are presented as red circles, green squares and black triangles, respectively. A dashed line was added to indicate a cut-off of Tg_stim,pre-I131_ = 1 ng/mL, highlighting the patients who had a Tg_stim,pre-I131_ below the cut-off value before radioiodine.

For a total of 84 patients, thyroid remnant imaging data were available for analysis. The median absorbed dose to the voxel with maximum uptake was 22.6 Gy (range <0.1–242 Gy) for 80 patients. In four patients, no thyroid remnant could be identified by a radiologist on the post-radioiodine whole-body or SPECT images. Figure 1 shows the maximum-voxel absorbed dose plotted against the post-operative stimulated Tg levels, excluding nine dosimetry patients who had positive Tg-Ab measurements. Of the 71 patients included for the dosimetry analysis who had negative Tg-Ab measurements, 13 patients had incomplete or indeterminate response, and 58 patients were classed as excellent responders. No correlation was found between maximum absorbed dose and post-operative stimulated Tg levels (*r* = −0.05, *P* = 0.68). Patients with incomplete response had on average a higher post-operative stimulated Tg, but no clear separation with respect to the maximum-voxel absorbed dose can be observed. In Fig. 1, a dashed line has been added to separate patients who had a post-operative stimulated Tg of less than 1 ng/mL, which, therefore, fulfilled the criteria of excellent response before radioiodine administration. All post-operative stimulated Tg measurements were performed within 14 days before radioiodine administration.

**Figure 2:**
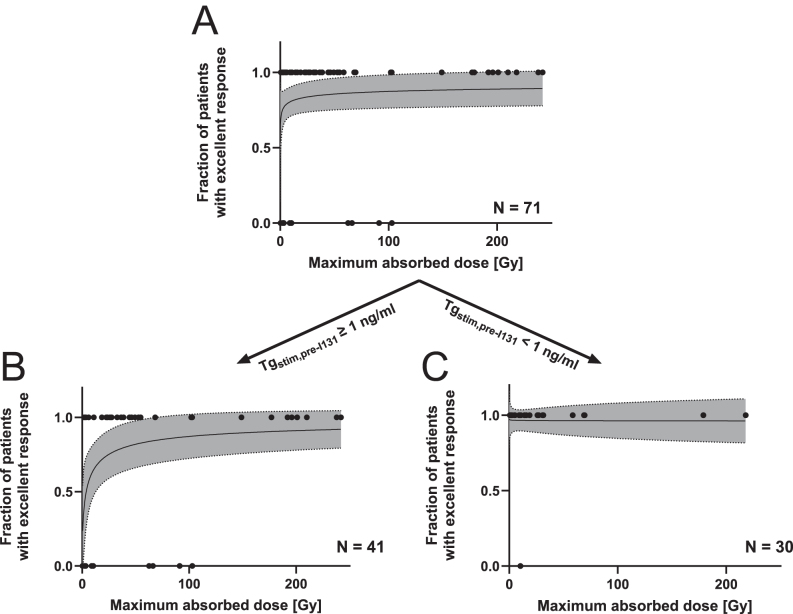
Patient outcome (1 = excellent response, 0 = indeterminate or incomplete response) as a function of maximum absorbed dose. Each patient is represented by an individual circle. (A) shows all patients with thyroid remnant dosimetry data, (B) only patients who had a Tg_stim,pre-I131_ ≥ 1 ng/mL and (C) only patients who had a Tg_stim,pre-I131_ < 1 ng/mL before radioiodine. Fitted dose–response relationships using a two-parameter log-logistic function have been added to the graphs, with the grey area highlighting the 95% confidence bands of the fit. Details of the fitted function used and the fit parameters can be found in the Supplementary Material.

Figure 2A shows the fraction of all patients with excellent response as a function of maximum absorbed dose. Figure 2B shows the fraction of patients with excellent response as a function of absorbed dose, but only including patients who had a Tg_stim,pre-I131_ ≥ 1 ng/mL. Figure 2C shows the fraction of patients with excellent response as a function of absorbed dose, but only including patients who had a Tg_stim,pre-I131_ < 1 ng/mL and therefore already fulfilled the criteria of excellent response before radioiodine exposure. The fitted dose–response relationships using a two-parameter log-logistic function have been added to Fig. 2 for illustration, including 95% confidence bands. Details of the fitting function used and the fit parameters are provided in Supplementary Table 4. Figure 2B shows that the response fraction increases with absorbed dose. The response fraction in Fig. 2C, on the other hand, shows no effect relationship with the absorbed dose delivered. To confirm these results, which were based on the fits to individual patient data, dose–effect relationships were also fitted with data binned into groups of *n* = 10 patients as a function of maximum absorbed dose. These additional fits are provided in Supplementary Fig. 1, showing very similar behaviour, with a dose–effect relationship best identifiable in the sub-analysis with patients Tg_stim,pre-I131_ ≥ 1 ng/mL.

### Statistical analysis

**Figure 3:**
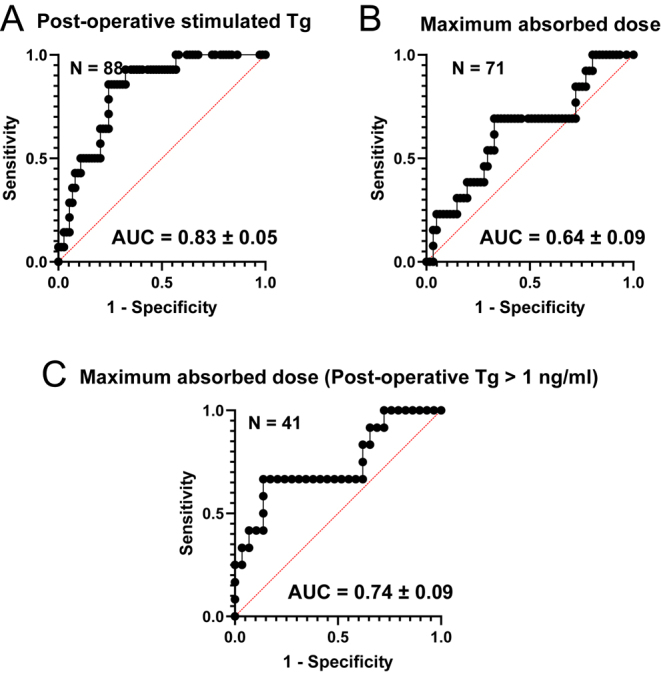
ROC curves for (A) post-operative stimulated Tg, (B) maximum absorbed dose to the thyroid remnant for all patients and (C) maximum absorbed dose to the thyroid remnant for patients with Tg_stim,pre-I131_ > 1 ng/mL, as predictors of excellent response at 9–12 months. An AUC of 0.5 would indicate the model performs no better than random guessing, while an AUC of 1.0 would represent perfect performance of the prediction model.

The results of the ROC analysis to predict excellent response are summarised in Table 3, while the ROC curves are presented in Fig. 3. An area under the curve (AUC) of 1.0 would indicate perfect performance of the prediction model, while an AUC of 0.5 would mean the model has no discernible predictive capability. The AUC of the maximum absorbed dose for all patients was not found to be statistically significantly different from 0.5. The ROC of post-operative stimulated Tg had an AUC of 0.83 (95% CI: 0.73–0.92). AUC for maximum absorbed dose for patients with Tg_stim,pre-I131_ ≥ 1 ng/mL was 0.74 (95% CI: 0.56–0.92). The AUC for post-operative stimulated Tg for patients with Tg_stim,pre-I131_ ≥ 1 ng/mL was 0.72 (95% CI: 0.59–0.85), see Supplementary Fig. 2.

**Table 3:** Results of the ROC analysis to predict ablation success using post-operative Tg and absorbed dose to the maximum voxel.

Variable	AUC	SE	95% CI	*P*-value
Tg_stim,pre-I131_	0.83	0.05	0.73–0.92	<0.001
D_max_	0.64	0.09	0.47–0.81	0.12
D_max_*	0.74	0.09	0.56–0.92	0.01

* Tg_stim,pre-I131_ ≥ 1 ng/mL.

SE, standard error.

## Discussion

Identifying predictive biomarkers for success of radioiodine therapy could potentially inform personalisation of treatment for individual patients. A stimulated post-operative thyroglobulin cut-off value of 1 ng/mL had previously been identified as a prognostic marker to identify patients at risk of a functional, structural or biologic event in the ESTIMABL2 clinical trial (16), and the results in this study support the case for post-operative thyroglobulin as a predictive biomarker (4, 5, 6). The results of this multi-centre, multinational prospective study show that stimulated post-operative Tg could potentially predict ablation success, and therefore inform personalisation of treatment (ROC AUC = 0.83).

Absorbed dose to the thyroid remnant should also correlate with ablation success (9, 11, 17). No dose–response relationship (Fig. 2C) could be identified in the subsets of patients with Tg_stim,pre-I131_ < 1 ng/mL before radioiodine, potentially indicating that radioiodine therapy makes no difference with respect to ablation success at 9–12 months for these patients. On the other hand, for patients with Tg_stim,pre-I131_ ≥ 1 ng/mL before radioiodine, the fraction of patients with excellent response at 9–12 months increased with the maximum absorbed dose (Fig. 2B). All patients with a maximum absorbed dose larger than 103 Gy were classed as excellent responders in the current study. The fitted dose–response relationship suggests that the response fraction at 9–12 months, for patients with Tg_stim,pre-I131_ ≥ 1 ng/mL before radioiodine, is 0.87 (95% CI: 0.72–1.01) and 0.91 (95% CI: 0.78–1.04) for 100 and 200 Gy maximum absorbed dose to the thyroid remnant, respectively. These findings should be replicated in a larger patient cohort and ideally with a longer follow-up time to assess the risk of recurrence in the two patient groups. The maximum absorbed doses to the thyroid remnants in all patients in this study are below the absorbed dose threshold of 300 Gy proposed in previous studies (18, 19), which could be explained by differences in the definition of treatment success, which consisted of negative follow-up imaging in previous studies.

A possible limitation of the current study was the use of single-time-point dosimetry in a subset of patients, which introduces potentially large uncertainties on the absorbed dose estimates (20). The calculations of the maximum absorbed dose, as originally proposed by Flux *et al.* (9), are relatively easy to implement but are only a surrogate of the absorbed dose to the total thyroid remnant. The calculations require a series of quantitative SPECT scans following radioiodine therapy. Acquisition of a single SPECT scan and application of a population half-life for the retention in the thyroid remnant is feasible, but will increase the uncertainty in the estimated absorbed doses and is not recommended if the results are used to inform treatment. In the present study, calculations of maximum thyroid absorbed dose required less than 1 h of medical physics time per patient. Nevertheless, cut-off values should be defined for specific populations and will vary between reconstruction and acquisition methodologies used at individual centres. The maximum absorbed dose might be an overestimation due to reconstruction artefacts, and may suffer from partial-volume effects dependent on the voxel size used. In addition, the maximum absorbed dose estimate has a large uncertainty in low-count scenarios with faint radioiodine uptake. Another limitation of the present study is the use of locally measured thyroglobulin levels, involving different Tg assays. It is well known that despite standardisation against human thyroglobulin reference material (CRM-457), there is considerable inter-assay variation (21). This may be a source of considerable heterogeneity in our results. The fact that we were nonetheless able to show the utility of post-operative Tg and maximum absorbed dose as biomarkers in response prediction only further supports that the effects observed in this study are of a clinically relevant magnitude.

The small number of ablation failures at 9–12 months is, while a good outcome for patients, a limitation of the study. To partially overcome this issue, indeterminate and incomplete response had to be combined to create the dose–response graphs and to assess these prognostic markers with respect to their ability to predict treatment success. Furthermore, follow-up imaging was not performed, and outcome at 9–12 months was solely based on Tg measurements. The relatively small number of treatment failures would require any future studies to include larger patient cohorts to allow for the extraction of further stratification factors for the treatment of DTC. The present work is therefore particularly important because it has shown that dose–response relationships can be identified even in a multi-centre, multinational study. Patient groups treated with 1.1 or 3.7 GBq could not be analysed separately due to the small number of 1.1 GBq patients enrolled. In addition, molecular testing is not part of standard practice in early-stage DTC and was not performed for these patients within this study. Therefore, information on molecular alterations, such as BRAF mutations, which may have implications for radioiodine uptake, could not be collected.

Following results of the ESTIMABL2 (16) and IoN (22) clinical trials, certain patients with low-risk DTC have been shown not to benefit from radioiodine ablation following thyroidectomy and will no longer be advised to undergo this treatment. This selected group of patients would now include patients enrolled in the current study. Nevertheless, the dosimetry results presented here contribute to our understanding of the utility of ablation and, in combination with post-operative Tg, may have a role in guiding future treatment decisions in those cohorts of patients who were excluded or were of insufficient numbers to draw conclusions (notably those with T3 and N1b disease) from the ESTIMABL2 and IoN trials. The dosimetry results presented here further support the findings of previous studies that patients with a post-operative stimulated Tg of <1 ng/mL likely do not benefit from radioiodine. In addition, the dose–response relationships suggest that individualising therapies for patients with Tg_stim,pre-I131_ ≥ 1 ng/mL should be further investigated.

## Conclusion

A dose–response relationship was observed for the maximum absorbed dose to the thyroid remnant in DTC patients when excluding patients with Tg_stim,pre-I131_ < 1 ng/mL before radioiodine. Patients with a post-operative stimulated Tg of <1 ng/mL do not appear to benefit from radioiodine. Both the maximum absorbed dose and the post-operative Tg are potential predictive factors to identify patients at risk of unsuccessful radioiodine ablation.

## Supplementary materials



## Declaration of interest

FAV has received speaker honoraria from Sanofi, AstraZeneca and Bayer (all fees paid to employer), as well as consultancy honoraria from GE Healthcare (fees paid to employer). MB supervises a PhD student sponsored by DOSIsoft. LVi has received honoraria from EISAI and AAA. FC has received honoraria from MAB, Novartis and AAA. MLa has received institutional grants from Novartis and Pentixapharm.

## Funding

The MEDIRAD project has received funding from the Euratom research and training programmehttps://doi.org/10.13039/100018708 2014–2018 under grant agreement No 755523. NHS funding was provided to the NIHR Biomedical Research Centre at The Royal Marsden and the ICR. The RTTQA group is funded by the National Institute for Health and Care Research (NIHR). We acknowledge infrastructure support from the NIHR Royal Marsden Clinical Research Facility Funding. This report is independent research funded by the NIHR. The views expressed in this publication are those of the author(s), and not necessarily those of the NHS, the NIHR or the Department of Health and Social Care.

## Author contribution statement

JT, IM, FL, KN, KHW, MLu, FAV, TS, LVi, FC, DV, MB, SS, UE, MLa and GF designed and/or performed the study. JT, HS, LVa, IM, PG, FL, CA and GF analysed the data. JT and PG performed statistical analysis. JT, IM, GF, KN and KHW wrote the first draft of the manuscript. All authors reviewed and approved the final manuscript.
