# Supplementary material for: Predictive factors of radioiodine ablation success: results from a MEDIRAD prospective clinical study for thyroid cancer
Source: Eur Thyroid J. 2025 Jul 1;14(4):e250097. doi: 10.1530/ETJ-25-0097 (PMC12229276; doi:10.1530/ETJ-25-0097)
Supplement: Supplementary file 1 [file supplementary_materials.pdf]

Supplementary material - Predictive factors of radioiodine ablation success: Results from a MEDIRAD prospective clinical study for thyroid cancer.

Supplementary Table 1: Ethics approval MEDIRAD WP3

Investigating centre	*Ethical approval obtained*	*Ethics approval details*	*National, EudraCT or ClinicalTrials.gov Identifier*
UMR & UKW	May 2019	The common study protocol was presented to the local ethics committees at the Medical Faculty of the University of Würzburg (UKW) and Marburg (UKM) and approved (UKW: Az. 246/18, UKM: Az. 83/19)	EudraCT: 2019-002244-25
IUCT-O	16th Dec 2019	Approved by the National Ethics Committee	ID RCB : 2019-A01734-53
RMH/ICR	19th March 2020	The study was approved by the East Midlands - Nottingham 1 Research Ethics Committee (20/EM/0022) and the institutional review board at the Royal Marsden Hospital	ClinicalTrials.gov: NCT04391244

Supplementary Table 2: Acquisition parameters used for ^{131}I imaging as part of the MEDIRAD WP3 study.

	^{131}I acquisition protocol
Collimator	High Energy
Photopeak energy window	364 keV ± 10% or ± 15%
SPECT(/CT) Matrix	128 x 128
SPECT movement	Body contour
Projections	2 x 30 (6° projection) or 2 x 36 (5° projection)
Time per projection	Adjusted based on measured count-rate for patient acquisition
CT	Standard low-dose protocol

Supplementary Table 3: SPECT (/CT) reconstruction parameters used for ^{131}I imaging as part of the MEDIRAD WP3 study.

	^{131}I reconstruction protocol
Reconstruction	OSEM (4 iterations, 10 subsets)
Attenuation correction (AC)	CTAC (one site: Chang with 0.11 cm^{-1} @ 364 keV)
Scatter correction	Triple-Energy Window (TEW)
Post-reconstruction filtering	None

Supplementary information on the fitted dose response relationships:

Fitting function used:

$$Y = \frac{1}{\exp\left(b \cdot \left(-e + \log\left(x\right)\right)\right) + 1}$$

Supplementary Table 4: Summary of the fit parameters of the dose-response relationship fit using a two-parameter log-logistic function shown in Figure 2 in the manuscript.

Fit parameter	*a) all patients with thryoid remnant dosimetry data*	*b) only including patients with* $\boldsymbol{Tg_{stim,pre-I131}} \geq \mathbf{1}\ \boldsymbol{ng/ml}$ *prior to radioiodine.*	*c) only including patients with* $\boldsymbol{Tg_{stim,pre-I131}} < \mathbf{1}\ \boldsymbol{ng/ml}$ *prior to radioiodine.*
Number of patients	71	41	30
Fit parameter - b	-0.4980	-1.350	0.07777
Fit parameter - e	-1.891	0.5775	44.21

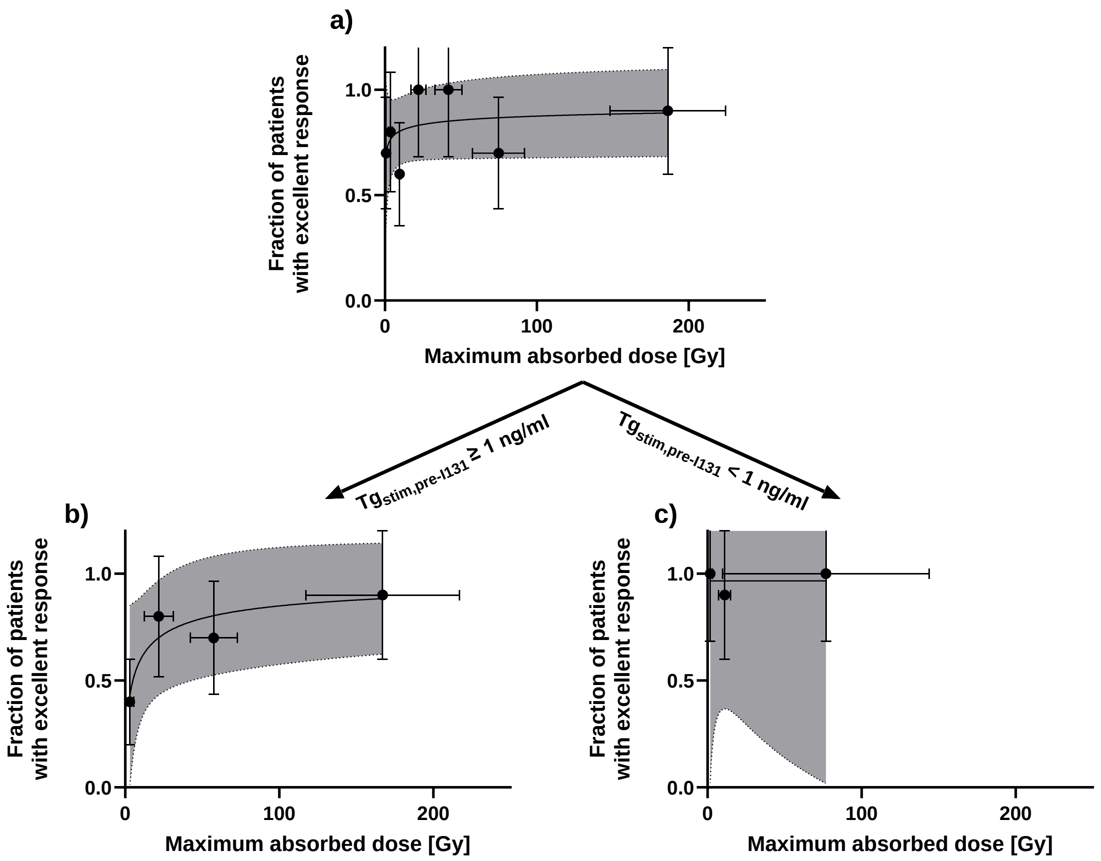

Supplementary Figure 1: Fraction of patients with excellent response with patients binned into groups of N=10 patients as a function of maximum absorbed dose for a) all patients with thryoid remnant dosimetry data, b) only including patients that had a $Tg_{stim,pre-I131} \geq 1\ ng/ml$ and c) only including patients that had a $Tg_{stim,pre-I131} < 1\ ng/ml$ prior to radioiodine. The mean of the maximum absorbed doses for each bin is shown together with the standard deviation. Uncertainties of fractions were estimated as $\sigma = \sqrt{N}$. Fitted dose-response relationships using a two-parameter log-logistic function have been added to both graphs with the grey area highlighting the 95% confidence bands.

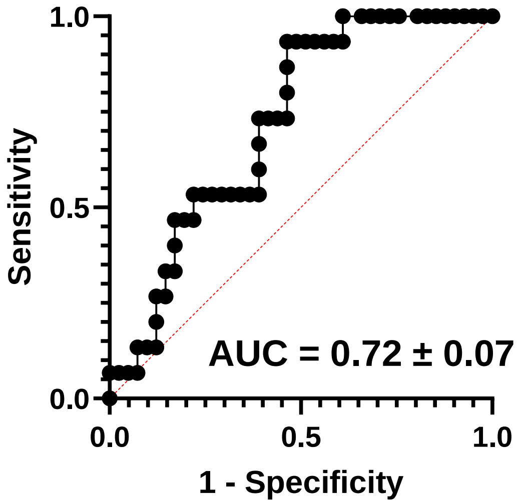

Supplementary Figure 2: ROC curves for post-operative stimulated Tg for patients with $Tg_{stim,pre-I131} > 1\ ng/ml$ as predictors of excellent response at 9-12 months.
